# Assessment of RT-qPCR Normalization Strategies for Accurate Quantification of Extracellular microRNAs in Murine Serum

**DOI:** 10.1371/journal.pone.0089237

**Published:** 2014-02-19

**Authors:** Thomas C. Roberts, Anna M. L. Coenen-Stass, Matthew J. A. Wood

**Affiliations:** 1 Department of Physiology, Anatomy and Genetics, University of Oxford, Oxford, United Kingdom; 2 Department of Molecular and Experimental Medicine, The Scripps Research Institute, La Jolla, California, United States of America; National Institutes of Health, United States of America

## Abstract

Extracellular microRNAs (miRNAs) are under investigation as minimally-invasive biomarkers for a wide range of disease conditions. We have recently shown in a mouse model of the progressive muscle-wasting condition Duchenne muscular dystrophy (DMD) that a set of highly elevated serum miRNAs reflects the regenerative status of muscle. These miRNAs are promising biomarkers for monitoring DMD disease progression and the response to experimental therapies. The gold standard miRNA detection methodology is Reverse Transcriptase-quantitative Polymerase Chain Reaction (RT-qPCR), which typically exhibits high sensitivity and wide dynamic range. Accurate determination of miRNA levels is affected by RT-qPCR normalization method and therefore selection of the optimal strategy is of critical importance. Serum miRNA abundance was measured by RT-qPCR array in 14 week old mice, and by individual RT-qPCR assays in a time course experiment spanning 48 weeks. Here we utilize these two datasets to assess the validity of three miRNA normalization strategies (a) normalization to the average of all Cq values from array experiments, (b) normalization to a stably expressed endogenous reference miRNA, and (c) normalization to an external spike-in synthetic oligonucleotide. Normalization approaches based on endogenous control miRNAs result in an under-estimation of miRNA levels by a factor of ∼2. An increase in total RNA and total miRNA was observed in dystrophic serum which may account for this systematic bias. We conclude that the optimal strategy for this model system is to normalize to a synthetic spike-in control oligonucleotide.

## Introduction

MicroRNAs (miRNAs) are abundant small RNA molecules that act as post-transcriptional regulators of gene expression in higher organisms. Two properties of miRNAs make them especially promising as extracellular disease biomarkers. Firstly, miRNAs act as master regulators of cellular pathways [1]. Therefore, by measuring miRNA expression, inferences can be made about pathophysiological processes (e.g. regeneration, inflammation, fibrosis) in the tissue of origin. Secondly, miRNAs exhibit remarkable stability in extracellular biofluids such as serum [2]–[4] on account of their association with proteins/lipoproteins [5]–[7] or by encapsulation within extracellular vesicles [8], [9]. Serum miRNAs have attracted much interest as biomarkers for a wide range of disease conditions, especially in the case of human malignancy.

The progressive muscle-wasting disorder Duchenne muscular dystrophy (DMD) is caused by loss-of-function mutations in the *DMD* gene, which encodes the dystrophin protein [10]. Experimental therapies for DMD, such as antisense oligonucleotide-mediated exon skipping to restore dystrophin protein, are in clinical trials [11], [12], although quantitative methods of measuring dystrophin restoration currently require muscle biopsy. There is therefore an urgent need for minimally-invasive biomarkers for the monitoring of disease progression and response to therapy.

Muscle diseases, such as DMD, are particularly well-suited for the study of extracellular miRNAs. In DMD, detecting a pathophysiologically relevant serum miRNA signal is trivial given that muscle comprises ∼50% of the total body mass. As a result, large fold-changes in serum miRNA levels can typically be detected in DMD patients and dystrophin-deficient animal models [13]–[15]. In contrast, the usefulness of serum miRNA biomarkers for cancer diagnostics has been questioned due to the low relative mass of tumors (e.g. it has been estimated that an ovarian tumor would need to grow for ∼8.8 years in order for tumor-derived miRNAs to be detectable in the circulation [16]). Similarly, Williams et al. recently showed that placenta-specific miRNAs are present in maternal serum at very low levels of (∼ 0.03 copies per ml) [17]. In contrast, models of DMD in which specific serum miRNAs are elevated ∼50–100 fold, are not subject to this limitation.

The gold standard methodology for quantifying miRNA expression is Reverse Transcriptase-quantitative Polymerase Chain Reaction (RT-qPCR). RT-qPCR is highly sensitive, specific and typically shows a dynamic range >6 logs. The small size of miRNAs means that they cannot be amplified by conventional RT-qPCR. Two commonly used detection strategies for detecting miRNAs are (a) gene-specific reverse transcription with a stem-loop primer followed by probe-based target detection [18] (e.g. Small RNA TaqMan), or (b) homopolymeric tailing of all miRNAs and intercalating dye-based target detection [19].

Conventional RT-qPCR studies in cultured cells or dissected tissues typically utilize the following general protocol. Reverse transcription is performed whereby the amount of input total RNA is standardized across samples. Gene-of-interest expression is then normalized to a reference (i.e. housekeeping) gene. This workflow is unsuitable for gene expression studies in biofluids (e.g. serum) for a number of reasons. Firstly, the relatively low RNA content (and high protein content) of serum make RNA quantification, and therefore standardization, difficult [20]. Furthermore, in order to maximize RNA extraction efficiency, some studies have utilized a protocol whereby bacteriophage or yeast RNA is added as a carrier at the phenolic extraction phase. Whilst maximizing RNA yield, this step makes RNA standardization between samples meaningless [21]. An additional complication is that there are no well-established serum reference genes (both for mRNAs and miRNAs).

We have recently reported extensive characterization of serum dystromiRs (dystrophy-associated microRNAs) in the *mdx* mouse model of DMD. Most notably we have profiled the serum miRNA content of wild-type, *mdx* and oligonucleotide treated-*mdx* mice at a single time point (14 weeks of age) and have followed the expression of three dystromiRs of interest (miR-1, miR-133a and miR-206) over a ∼12 month period [22] (datasets are summarized in **Table S1**). In the present study we utilize these datasets to systematically investigate three potential solutions to the problem of normalization in miRNA RT-qPCR experiments in serum. (a) Normalization to the average Cq value of all measured mRNA assays. This strategy is useful in genomics experiments when there is an absence of *a priori* knowledge of stable reference genes but is necessarily restricted to experiments in which large numbers of miRNAs are assayed [23]. (b) Normalization to candidate endogenous reference miRNAs. In this study we investigate the use miR-16, miR-31 and miR-223 as reference (housekeeping) miRNAs. These miRNAs were included because miR-16 is commonly used as a reference miRNA in cell and tissue studies [15], [24], miR-223 has been previously used as a normalizer by our group [15] and others [13], [25], and miR-31 has been recently been proposed as a novel normalizer [26]. (c) Normalization to an external spike-in RNA control introduced during the phenolic phase of the RNA extraction protocol.

The key findings of our previous studies are unaffected by choice of normalization strategy due to the large effect sizes which are typically measured [15], [22]. However, choice of normalization strategy becomes much more important when trying to quantify miRNAs in a more outbred context (i.e. in DMD patients) where ‘noisy’ genetic background may obscure biologically significant changes in miRNA levels, and in the case of miRNAs which exhibit smaller changes in expression or are less abundant (e.g. in the case of miRNAs released from tumors or in other more localized disease processes). We conclude that the optimal strategy for quantifying extracellular miRNAs in dystrophic serum is to normalize to an external RNA spike-in control as we show that the total RNA and total miRNA content of serum is an experimental variable and that normalization to candidate endogenous control miRNAs introduces a systematic bias in miRNA quantification.

## Materials and Methods

### Animal Experimentation

Ethical approval for animal experimentation was initially approved by the University of Oxford’s Local Ethical Review Committee. All animal procedures described here are permitted under PPL 30/2907 awarded to Professor Matthew J.A. Wood at the University of Oxford by the UK Home Office in accordance with UK law (the Animals [Scientific Procedures] Act 1986). Animals were housed in standard cages and all procedures were performed at the Biomedical Sciences Building (BSB) at the University of Oxford. Animal welfare was regularly monitored by researchers and BSB veterinary staff. The experimental datasets used for the analyses in this study have been described in detail previously [22] (**Table S1**). Briefly, 12.5 mg/kg of Peptide Phosphorodiamidate Morpholino Oligonucleotide (PPMO, either Pip6e-PMO or Pip6a-PMO [27]) was administered via the tail vein of 12 week old male *mdx* mice under isoflurane anaesthesia. C57/Bl10, *mdx* or PPMO treated *mdx* mice were harvested by rising CO_2_ concentration and cervical dislocation at appropriate time points.

### RNA Extraction

Animals were sacrificed and whole blood collected from the jugular vein using Microvette CB300 capillary serum collection tubes as directed by the manufacturer (Sarstedt Ltd, Leicester, UK). RNA was extracted from 50 µl of serum using TRIzol LS reagent (Life Technologies, Paisley, UK) as according to manufacturer’s instructions with minor modifications. The *Caenorhabditis elegans* miRNA cel-miR-39 (5′-UCACCGGGUGUAAAUCAGCUUG) was used as a synthetic spike-in control RNA oligonucleotide (IDT, Leuven, Belgium) as it has no mammalian homologue [28], [29]. After addition of TRIzol LS reagent, 3 µl of 5 nM synthetic miRNA oligonucleotide [29] was added and the samples vortexed vigorously to ensure complete mixing [2]. 20 µg of RNase-free glycogen (Roche, Burgess Hill, UK) was used as carrier to improve extraction efficiency. RNA was re-suspended in 30 µl of nuclease-free water. Where appropriate, serum total RNA was quantified using the Quant-iT RiboGreen RNA Assay Kit (Life Technologies).

### Small RNA TaqMan RT-qPCR

All RT-qPCR studies were designed to comply with the MIQE guidelines where applicable or practical [30], [31] (**File S1**). Small RNA TaqMan Assays (Life Technologies) were performed as according to manufacturer’s instructions as described previously [22]. Serum RNA samples were reverse transcribed using the TaqMan MicroRNA Reverse Transcription Kit (Life Technologies) using miRNA-specific stem-loop RT. qPCR analysis was performed on a StepOne Plus real-time thermocycler using TaqMan Gene Expression Mastermix (Life Technologies). All primer/probe assays are listed in **Table S2**. [32]. TaqMan assays were validated by performing serial dilutions of cDNA to produce standard curves in order to demonstrate assay linearity and dynamic range (**Fig. S1**). PCR efficiencies were determined by linear regression analysis performed directly on the sample data using LinRegPCR [33] (**Table S2**). Data were analyzed using the Pfaffl method in order to correct for PCR efficiency [32].

### miRCURY RT-qPCR Array

Serum miRNA profiling was performed on the miRCURY LNA SYBR Green RT-qPCR array platform by Exiqon Services (Copenhagen, Denmark) as described in detail previously [22]. 15 µl of RNA was reverse transcribed in 75 µl reactions using the miRCURY LNA Universal RT microRNA PCR, Polyadenylation and cDNA synthesis kit (Exiqon). cDNA was diluted 1 in 50 and assayed in 10 µl PCR reactions on the Rodent panel I and panel II arrays. RT-qPCR was performed on a LightCycler 480 Real-Time PCR System (Roche). No template controls (NTCs) and intra-plate controls (IPCs) are given in **Fig. S2** and **Fig. S3** respectively.

### Statistical Analysis

miRNA expression stability was determined using the Normfinder Visual Basic application for Microsoft Excel [34]. Correlation analyses, one-way analysis of variance (ANOVA) or two-way ANOVA (with Bonferroni *post hoc* tests) as appropriate (GraphPad Software Inc, La Jolla, CA, USA).

## Results

### Comparison of Normalizer Stability

**Figure 1:**
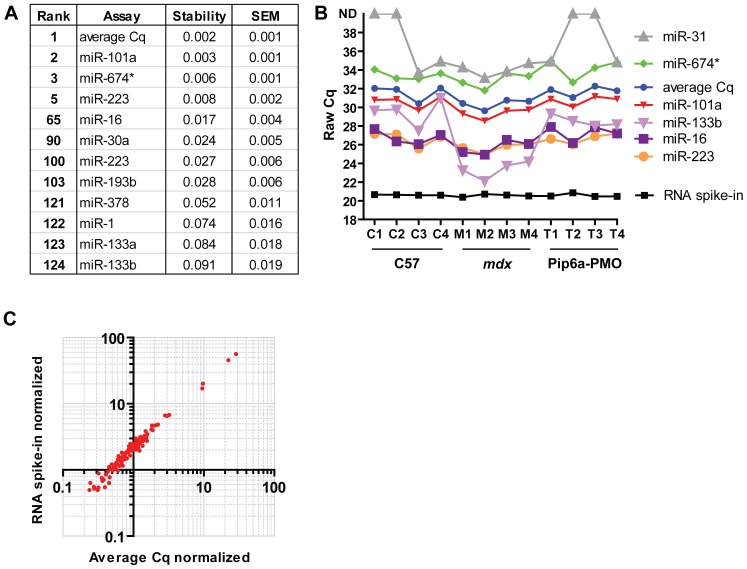
Effect of normalization strategy on global serum microRNA abundance. (A) Key results from NormFinder algorithm. The most stable normalizer (the average of all Cq values) is ranked as 1, the least stable (miR-133b) is ranked as 124. Stability scores and standard error of the mean are shown. (B) Raw Cq data for candidates reference miRNAs and RNA spike-in control. High ranked NormFinder candidates are shown. miR-133b which is known to be highly up-regulated in *mdx* serum is shown for comparison. (C) C57 vs *mdx* expression ratios as determined by the average Cq method (x-axis) or normalized to the RNA spike (y-axis).

By performing serum miRNA profiling on the miRCURY LNA SYBR green RT-qPCR array platform we were able to directly assess three different normalization strategies. The arrays consist of 741 RT-qPCR assays of which 124 were detected in all samples (**Table S1**). The NormFinder algorithm [34] was used to determine the relative stabilities of those miRNAs that were detected in all samples (**Fig. 1A**). The highest ranked normalizer was the average Cq of all 124 assays (mean Cq = 31.23, SD = 0.85, n = 12), with the most stable single miRNAs being miR-101a and miR-674*. As expected, dystromiRs which are known to be up-regulated in *mdx* mouse serum (i.e. miR-1 and miR-133) were the least stable. miR-223, was found to rank highly (5^th^ most stable). Conversely, miR-16 has a lower stability value (ranking 65^th^). miR-31 stability was not calculated as it was not detected in all samples. An external spike-in control oligonucleotide was added during sample preparation in order to monitor the efficiency of RNA extraction and to determine if PCR inhibitors were co-purified with serum RNA. Detection of the RNA spike-in oligonucleotide was highly consistent between samples (mean Cq = 20.58, SD = 0.13, n = 12) indicating equivalent RNA extraction efficiencies. The relative stabilities of raw Cq data for individual miRNAs of interest and the RNA spike-in oligonucleotide are shown in **Fig. 1B**. Data for miR-133b, which is differentially abundant in *mdx* serum, is shown for comparison. Furthermore, raw data for miR-31 is also shown as it has recently been suggested that the dystromiR/miR-31 ratio is a useful method of distinguishing disease and healthy animals [26]. However, miR-31 was expressed at very low levels and not detected in all samples. Comparison of the raw Cq data shows that abundance of the RNA spike-in oligonucleotide is the most consistent, suggesting that this may be a useful normalizer. Interestingly, the average Cq value correlated well with both miR-16 (r = 0.890, *P* = 0.0001) and miR-223 (r = 0.959, *P* = 0.000001) suggesting that normalization to either of these miRNAs will likely produce similar results to normalizing to the average Cq of all assays.

### Effect of Different Normalization Strategies on Results

To determine the effect of different normalization strategies on the array results, the data were analyzed by both the average Cq method and the external RNA spike-in control method. C57 vs *mdx* expression ratios for each normalization method were calculated and plotted against each other (**Fig. 1C**). The resulting plot shows that the expression ratios for all 124 miRNAs are shifted upwards with respect to the origin, indicating that expression ratios were called as higher when the data were normalized to the external RNA spike-in control. The fold increase relative to the average Cq method ranged from 1.39 to 2.77 fold (mean = 2.15 fold increase, SD = 0.26, n = 124). These data suggest that the choice of normalization method can influence the calculated expression ratios by a factor of ∼2 in this experimental system.

**Figure 2:**
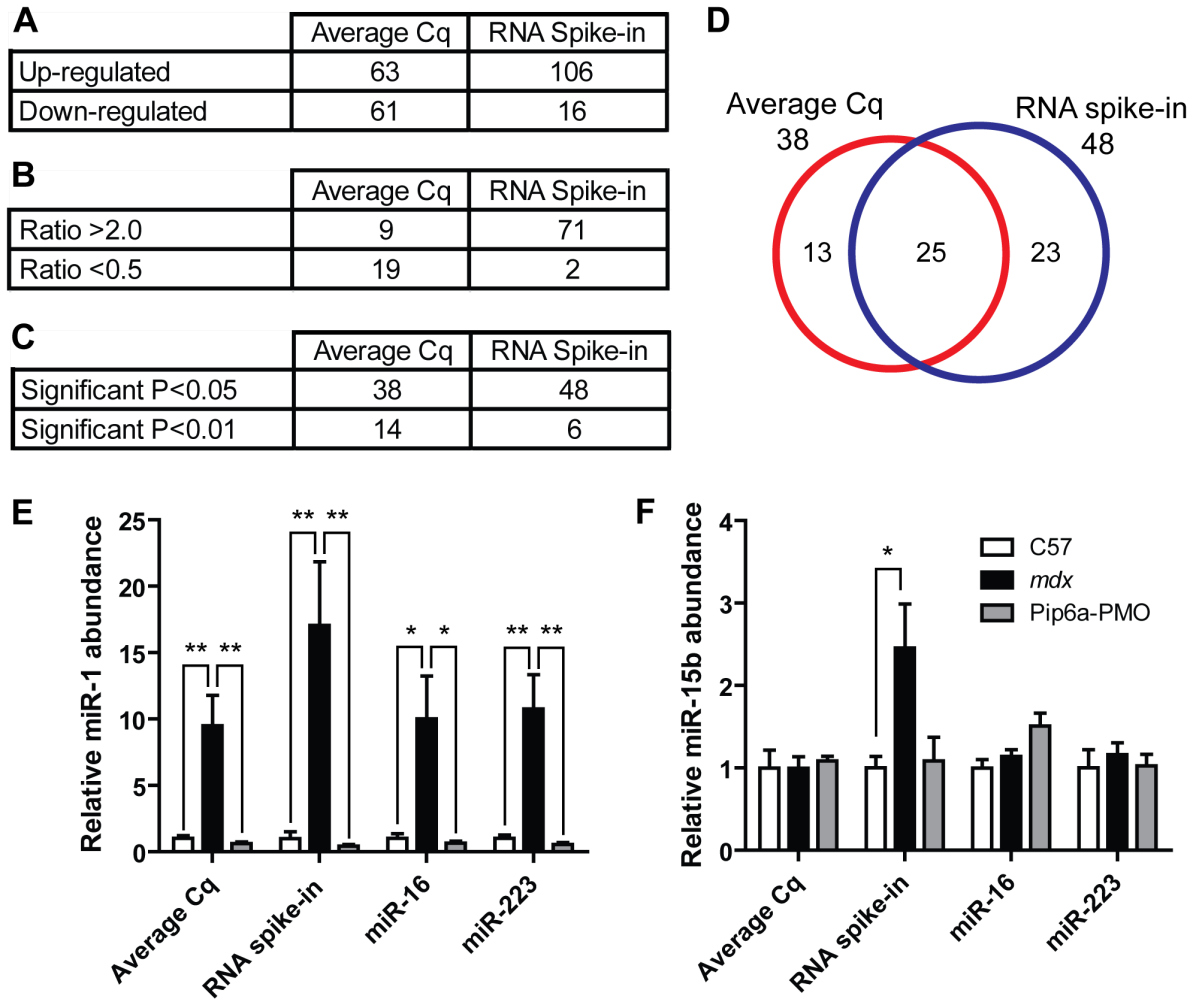
Effect of normalization strategies on individual microRNA expression. Comparison of the average Cq and RNA spike-in normalization strategies in terms of (A) number of up- and down-regulated miRNAs, (B) number of miRNAs with expression ratios greater than 2 or less than 0.5, and (C) number of statistically significant miRNAs. (D) Venn diagram showing overlap between statistically significant miRNAs as determined by both normalization methods. Relative serum miRNA were determined for individual miRNAs analysed on the RT-qPCR array for C57, *mdx* and Pip6a-PMO treated *mdx* samples. The effects of normalization to the average Cq, RNA spike control, miR-223 or miR-16 are shown for (E) miR-1, (F) miR-15b. All values are mean+SEM, *P<0.05, **P<0.01.

The ratio of up- and down-regulated genes for the average Cq method was approximately 1∶1, whereas the RNA spike-in method called many more miRNAs were called as up-regulated than were down-regulated (**Fig. 2A**). The number of miRNAs with expression ratios above 2 also increased in the RNA spike-in normalized data set (**Fig. 2B**) as did the number of statistically significant changes (**Fig. 2C**). 25 common miRNAs were statistically significant as determined by both normalization strategies, while 13 and 23 were unique to the average Cq and RNA spike-in data sets respectively (**Fig. 2D**).

The differences between normalization methods did not fundamentally change the interpretation of our previous results for miRNAs which show large, statistically significant differences in expression such as miR-1 (**Fig. 2E**) (although the significance was lower when normalizing to miR-16). The situation was different for miRNAs with lower differential expression ratios. For example, miR-15b was not significantly changed (0.99 fold) in *mdx* serum by the average Cq method but was significantly changed 2.46 fold (*P* = 0.0319) when expression was normalized to the external RNA control (**Fig. 2F**). As expected, normalization to miR-16 and miR-223 produced similar expression ratios to the average Cq method.

### Total miRNA and Total RNA are Increased in *mdx* Serum

**Figure 3:**
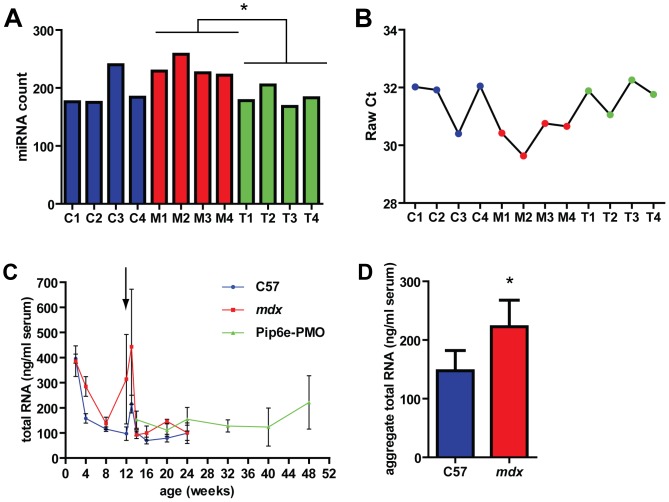
Total microRNA levels and total RNA levels are increased in *mdx* serum. Data from miRCURY serum miRNA profiling suggests an increase in total miRNA in *mdx* samples as (A) the number of miRNAs detected was generally higher in the *mdx* samples (*p<0.05, 2-tailed student’s t-test) and, (B) mean raw Cq values were generally lower in *mdx* samples indicating higher abundances. (C) Total serum RNA was determined by RiboGreen assay for C57, *mdx* and Pip6e-PMO treated *mdx* over a series of available time points. Values are mean+SEM, n = 3–5. Arrow indicates time of injection (single intravenous 12.5 mg/kg dose Pip6e-PMO). (D) Aggregate total serum RNA over the range of 2–24 weeks of age. Values are mean+SEM, n = 9, *P<0.05, two-way ANOVA.

Interrogation of the miRNA RT-qPCR array dataset revealed that generally more miRNA assays were called as detected in the *mdx* samples than the other groups (**Fig. 3A**), and the average Cq of all assays was generally lower in the *mdx* samples (**Fig. 3B**) suggesting an increase in the total amount of miRNA in dystrophic serum. We have previously measured serum miRNA abundance in C57, *mdx* and Pip6e-PMO treated *mdx* mice at regular time points over a ∼12 month period [15] (**Table S1**). In order to determine if the amount of total RNA increases in the serum of *mdx* mice, serum RNA samples from the time course study were analyzed by RiboGreen assay. Total RNA was generally higher in the *mdx* samples (**Fig. 3C**). Aggregate mean serum RNA values from all comparable time points showed that total RNA amounts were significantly higher in *mdx* serum relative to C57 serum (*P* = 0.0237, two-way ANOVA) (**Fig. 3D**).

### Stability of Various Control miRNAs Over Time

**Figure 4:**
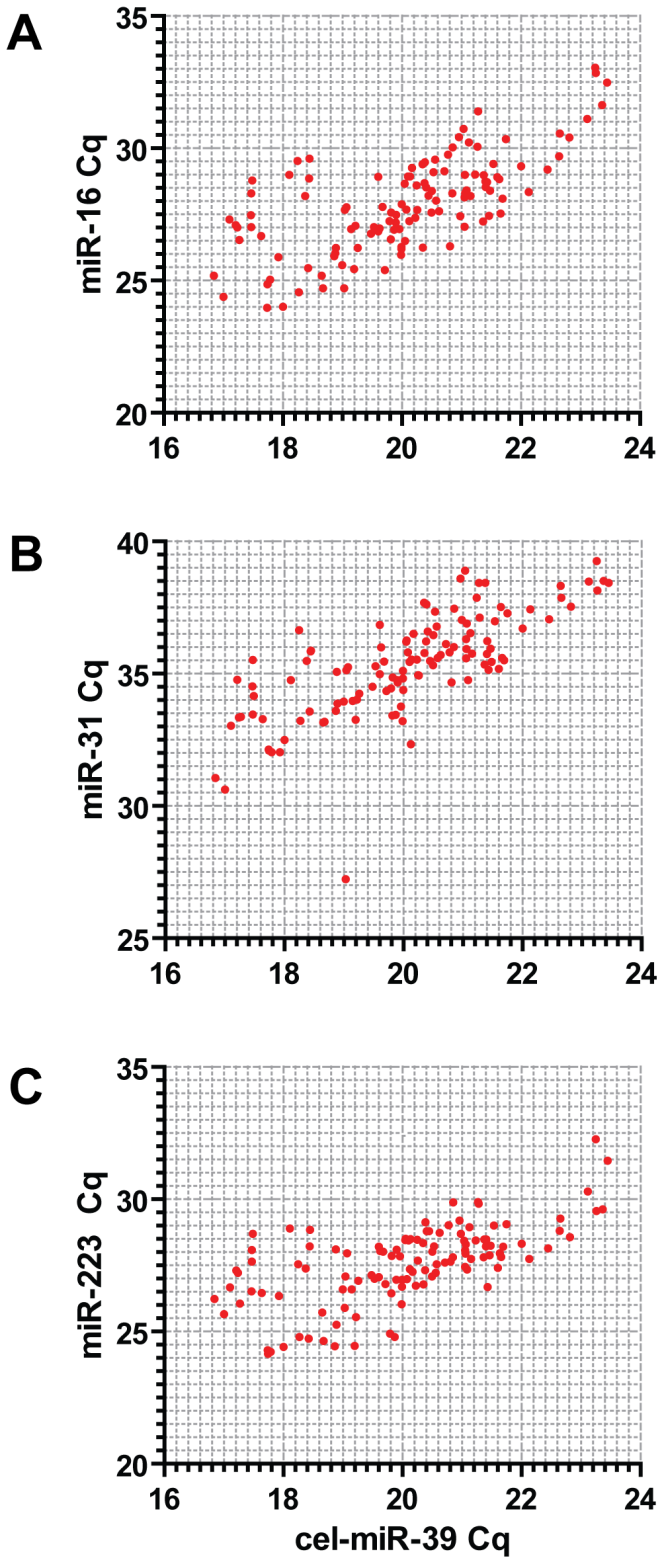
Correlation of Cq values between endogenous miRNAs and the external spike-in control for time course samples. Plot of raw Cq values from the time course study for cel-miR-39 against (A) miR-16, (B) miR-31, and (C) miR-223.

**Figure 5:**
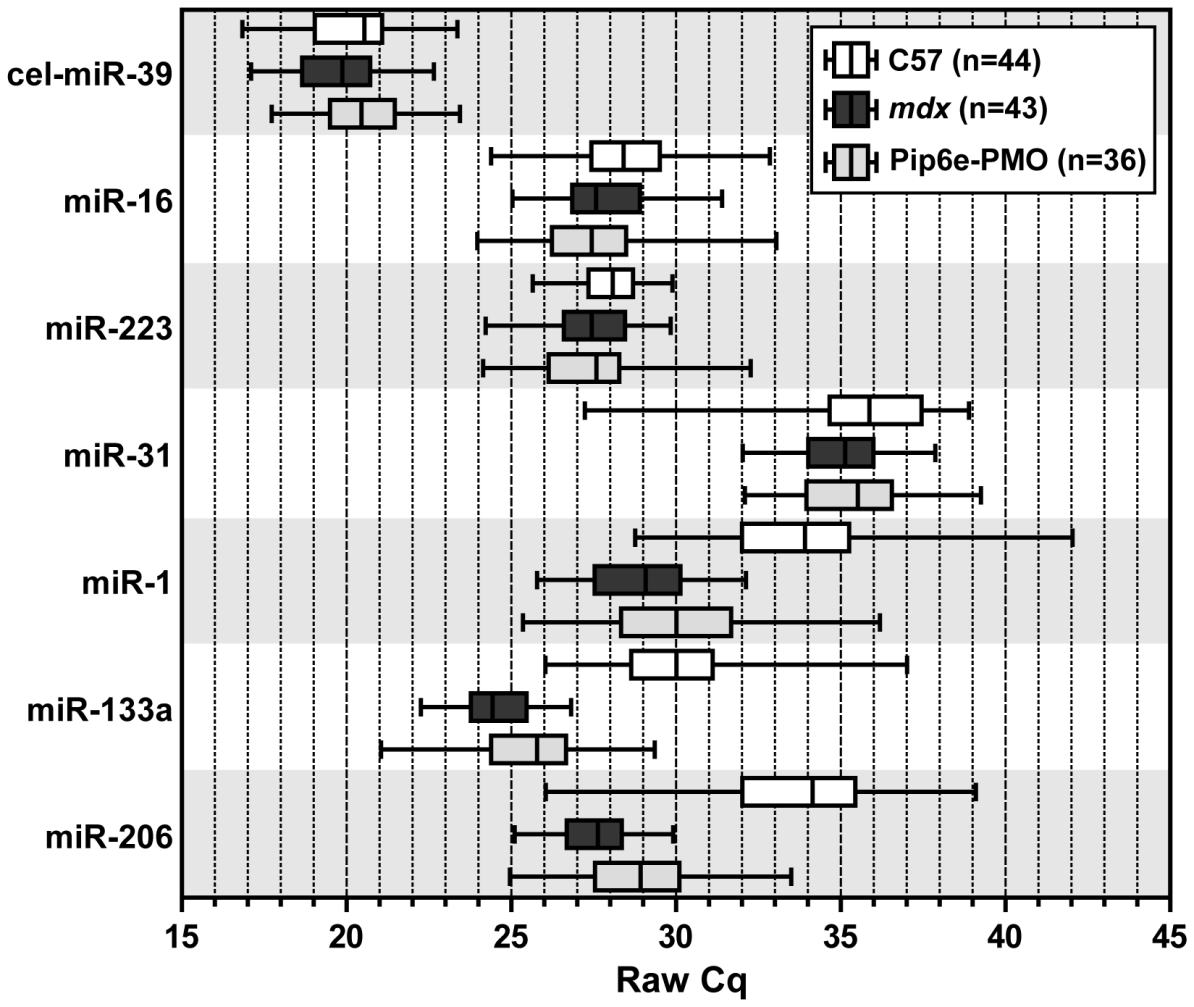
Aggregate microRNA raw Cq data. Tukey box plots showing raw Cq data aggregated over all time points and separated by experimental condition for each miRNA assayed by small RNA TaqMan RT-qPCR. n numbers for each experimental group are indicated.

In order to further assess the use of miR-16, miR-31 and miR-223 as reference miRNAs we performed individual small RNA TaqMan assays on the same serum RNA samples used in the time course study (**Table S1**). Pre-normalization raw Cq data for miR-16, miR-31 and miR-223 were plotted against the spike-in control (cel-miR-39) for each individual sample over all time points measured (**Fig. 4**). All three endogenous miRNAs showed positive and highly significant correlations with the spike-in control (miR-16: r = 0.691 and *P* = 5×10^−18^, miR-31 r = 0.734 and *P* = 3.2×10^−21^, miR-223 r = 0.637 and *P* = 8.5×10^−15^). These data suggest that normalizing to the spike-in control effectively accounts for differences in RNA extraction efficiency while being independent of shifts in global miRNA levels. To further assess the variation in miRNA abundance in the time course study samples, raw Cq data for each assay were aggregated over all time points analyzed and represented in Tukey box plots for C57, *mdx* or PPMO treated *mdx* samples (**Fig. 5**). These data show that detection of the external spike-in control miRNA (cel-miR-39) is highly stable (mean Cq = 20.09, Cq SD = 1.53, n = 123) over all time points measured suggesting comparable RNA extraction efficiencies between all 123 samples included in this study. cel-miR-39 and miR-223 exhibited the least variation over all time points (Cq SD = 1.53 and 1.48 respectively) although miR-223 was statistically higher in the *mdx* samples measured over the whole time course (*P* = 0.0128).

**Figure 6:**
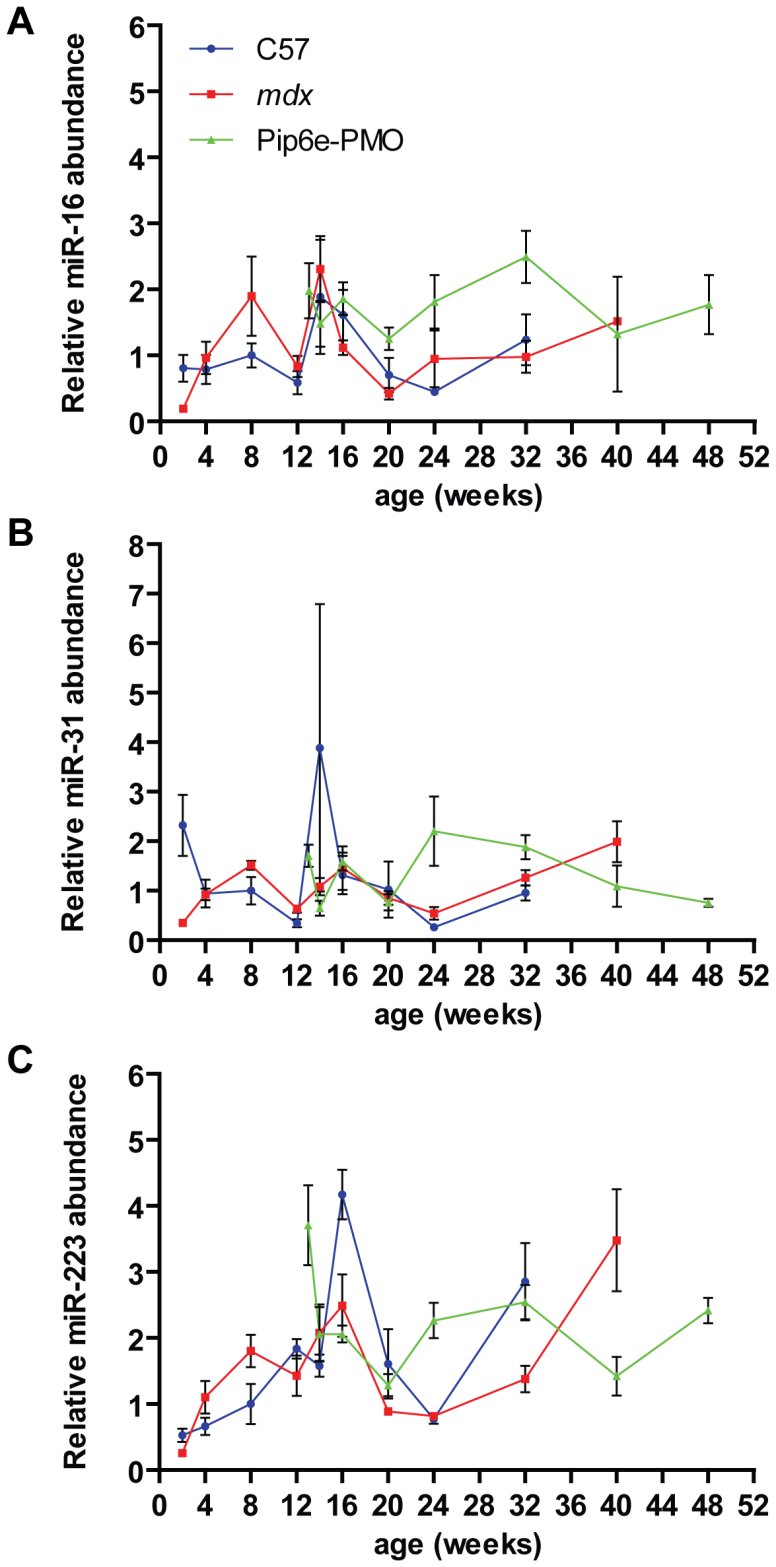
Serum time course of putative reference miRNA abundance. Male C57Bl/10, *mdx* and Pip6e-PMO treated *mdx* mice were sacrificed at various ages and serum miRNA levels determined by small RNA TaqMan RT-qPCR. (A) miR-1, (B) miR-133a and (C) miR-206 abundance was normalized to an external spike-in control. All miRNA expression data were normalized to the mean of the 8 week old C57Bl/10 group. Values are mean +/− SEM, n = 3–8.

Subsequently, all data were normalized to the external RNA spike-in control and the mean miRNA abundance for each experimental condition and time point plotted. Levels of the reference miRNAs in all three experimental groups showed fluctuations over the various time points indicating relatively poor stability between experimental groups (**Fig. 6**). In general, all three miRNAs were slightly elevated in *mdx* serum. However, at 2 weeks of age the reverse was true with all three miRNAs showing higher abundances in C57 serum. Similarly, miR-31 and miR-223 also show spikes in the C57 samples at 14 and 16 weeks respectively. The changes observed between C57 and *mdx* samples are insufficient to explain the large changes in dystromiR abundance for several reasons. Firstly, the changes in the putative reference genes occur at different time points to the peaks in dystromiR abundance that we have reported previously [22]. Secondly, the effect sizes for these changes (∼2–4 fold) are much lower than are observed with the dystromiRs (∼50–100 fold). This is consistent with a general increase in total miRNA levels.

## Discussion

In this study we have investigated the use of different normalization strategies for the quantification of extracellular miRNAs in mouse serum. The levels of the putative endogenous miRNAs were positively correlated with the external spike-in control, indicating that variations in the latter reflect changes in endogenous miRNA abundance. Furthermore, the external spike-in control was the least variable miRNA across all samples in the RT-qPCR array study (**Fig. 1B**), and between experimental groups in the time course study (**Fig. 5**) thus validating this normalization approach. Additionally, more miRNAs were called as statistically significant, and greater fold changes observed, when the data were normalized to the spike-in control as opposed to the average Cq (**Figs. 2B,C**). The ratio of up- and down-regulated miRNAs was more balanced when the data were normalized to the average Cq (**Fig. 2A**). However, the observation of a global shift in miRNA levels suggests this ‘balance’ is artificial (**Fig. 3**). Together, these data support the use of an external spike-in RNA normalization strategy.

Conversely, normalization to an endogenous miRNA presents a number of problems when comparing C57 and *mdx* serum samples. Analysis of the array data reveals a systematic ∼2 fold difference in expression ratio when the data are normalized to an external spike-in RNA control compared with methods that normalize to endogenous serum miRNA(s) (**Figs. 1C** **,** **2E,F**). This difference can be explained, at least in part, by an increase in total RNA/total miRNA in dystrophic serum. The ∼2 fold increase in total serum RNA (**Fig. 3D**) corresponds well with the ∼2 fold difference in expression ratio observed between the different normalization strategies. Importantly, normalization strategies based on the abundance of endogenous miRNAs assume that the global miRNA content of each sample is approximately equivalent. Given that total serum RNA and miRNA is a dependent variable in this experimental system, normalization to an endogenous miRNA(s) will inevitably lead to quantification errors. Consequently, expression ratios determined by endogenous miRNA-based normalization methods will fail to account for a global increase in miRNA levels in dystrophic serum. As a result, fold changes calculated by this method are likely conservative and will tend to under-estimate miRNA abundance in dystrophic serum samples. As a result, the detection of some small magnitude fold-changes is dependent on the normalization strategy used (**Fig. 2F**). We have also observed considerable natural variation in the abundance of the proposed endogenous control miRNAs (miR-16 and miR-223) with these miRNAs showing variable expression across the time course samples, and between experimental groups (**Figs. 5** **,** **6**). It remains to be seen if a global increase in miRNA content is observed in other pathological conditions or is specific to dystrophic serum. Consequently, in the absence of *a priori* knowledge of endogenous miRNA stability across all experimental conditions, normalization to an external control is advisable.

Recently, Vignier et al. have reported a decrease in miR-31 abundance in the serum of DMD patients and *mdx*-5CV mice and suggested that miR-31 can be used as a normalizer for dystromiR abundance [26]. The data reported in the present study, and our previous work [15], [22], do not support this notion. Firstly, we have previously reported that miR-31 is moderately elevated in the serum of 8 week old *mdx* mice [15] and not decreased as reported by Vignier et al. Similarly, by performing miRNA profiling on 14 week old mice we again report here that miR-31 shows a tendency to increase in *mdx* serum samples and was not detected in several of the C57 and treated samples (**Fig. 1B**). Furthermore, miR-31 is also found to be elevated in DMD patient serum (Francesco Muntoni, personal communication). These simple observations are sufficient to invalidate the ‘ratio-to-miR-31’ method. Analysis of miR-31 over a ∼12 month period reveals further problems for its utility as a normalization control. miR-31 was generally found to be increased in *mdx* samples (at 8, 12, 16, 24 and 32 weeks of age) but decreased at others (**Fig. 6B**). The fold changes were generally small and are probably within the natural biological variation in circulating miR-31 levels. Importantly, miR-31 is present at very low levels in mouse serum (average Cq = 35.4, SD = 1.92) (**Fig. 5**), which is approaching the lower limit of reliable quantification by RT-qPCR methods (and explains why it was called as ‘not detected’ in some samples in our array profiling study [**Fig. 1B**]). In summary, multiple lines of evidence suggest that miR-31 is unsuitable for normalization of serum miRNAs in dystrophic serum.

This work presents a rationale for standardizing serum volume between experimental samples rather than standardizing RNA input at the reverse transcription stage. Indeed, standardization of serum volume is standard practice for conventional clinical biochemistry assays. Normalization to an external spike-in control will thus give a result that more accurately reflects the absolute number of miRNA copies per volume and is unaffected by shifts in global miRNA content. Although the key findings of previous studies (which have focused on the miRNAs: miR-1, −133 and −206) are unaffected by the strategy used, optimal normalization will be essential for quantifying miRNAs in a more genetically outbred patient population, and in the case of miRNAs with smaller changes in serum abundance. In contrast with other studies which have focused on endogenous reference miRNAs [35]–[38] we conclude that normalization to an external spike-in oligonucleotide is the optimal strategy for accurate determination of miRNA abundance in dystrophic serum.

## Supporting Information

Figure S1 **RT-qPCR Validation.** Standard curves demonstrating linearity and dynamic range of Small RNA TaqMan assays used in this study for (A) miR-1, (B) miR-133a, (C) miR-206, (D) cel-miR-39, (E) miR-16, (F) miR-31, and (G) miR-223.
(TIF)

Figure S2 **No template control signals for miRCURY array assays.** Undetected samples are given the Cq value 40. Positive spike-in controls were included in the water bank sample and are shown on the right of the figure.
(TIF)

Figure S3 **Intra-Plate Controls.** Raw Cq values for two intra-plate controls (IPC1 and IPC2) over all samples. The average of all Cqs is shown for comparison.
(TIF)

Table S1 Summary of datasets used in this study. *single 12.5 mg/kg intravenous dose of Pip6a-PMO which induces efficient dystrophin restoration. **single 12.5 mg/kg intravenous dose of Pip6e-PMO which induces efficient dystrophin restoration.
(DOCX)

Table S2 List of RT-qPCR Assays used in this study. Assay ID, PCR efficiencies determined using LinRegPCR and R^2^ values from standard curves are shown.
(DOCX)

File S1 **MIQE Compliance Information.**
(DOCX)

Checklist S1 **ARRIVE guidelines checklist.**
(DOC)
