# Supplementary figures and images for: Assessment of RT-qPCR Normalization Strategies for Accurate Quantification of Extracellular microRNAs in Murine Serum
Source: PLoS One. 2014 Feb 19;9(2):e89237. doi: 10.1371/journal.pone.0089237 (PMC3929707; doi:10.1371/journal.pone.0089237)

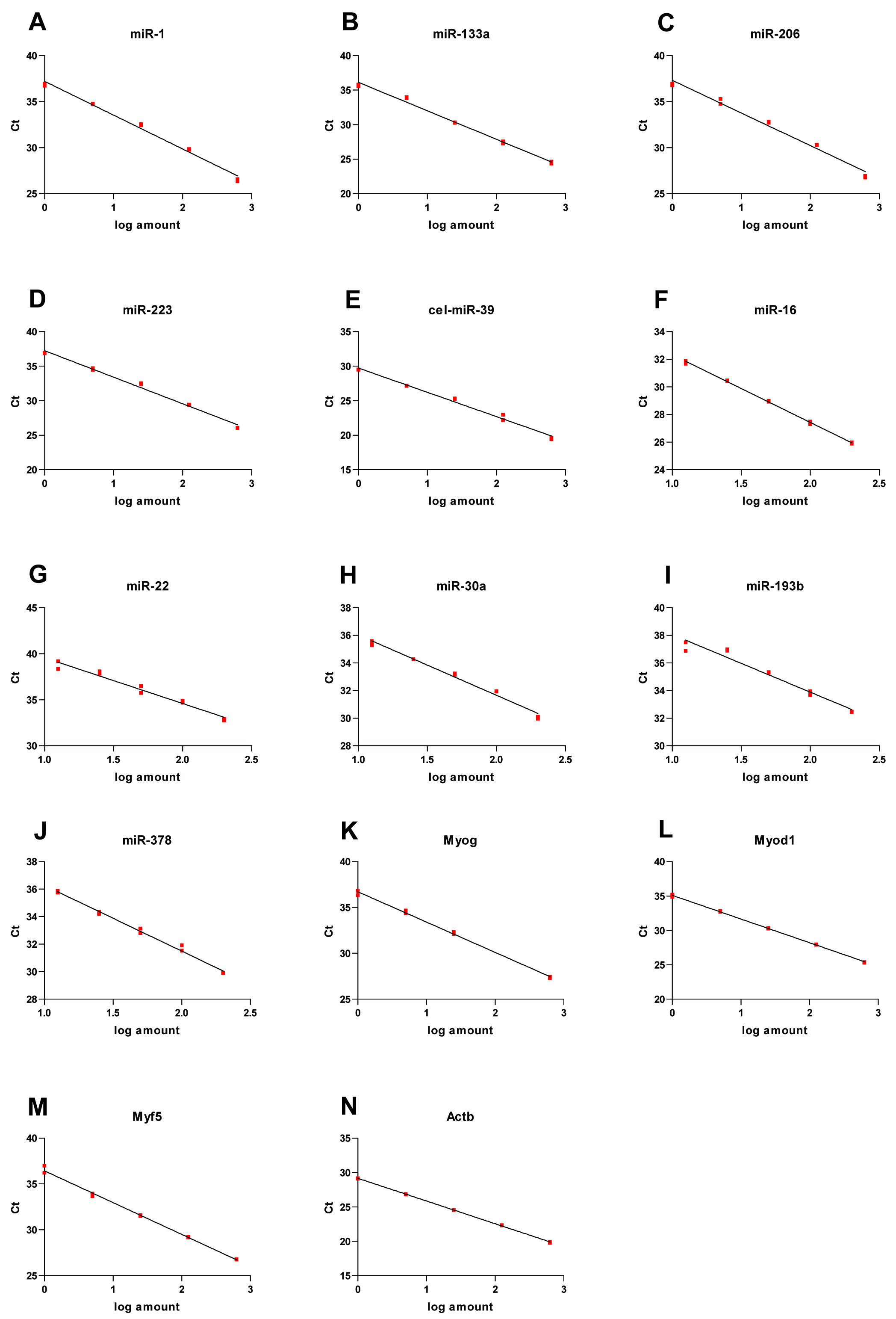

Supplement: Figure S1 — RT-qPCR Validation. Standard curves demonstrating linearity and dynamic range of Small RNA TaqMan assays used in this study for (A) miR-1, (B) miR-133a, (C) miR-206, (D) cel-miR-39, (E) miR-16, (F) miR-31, and (G) miR-223. (TIF) [file pone.0089237.s001.tif]

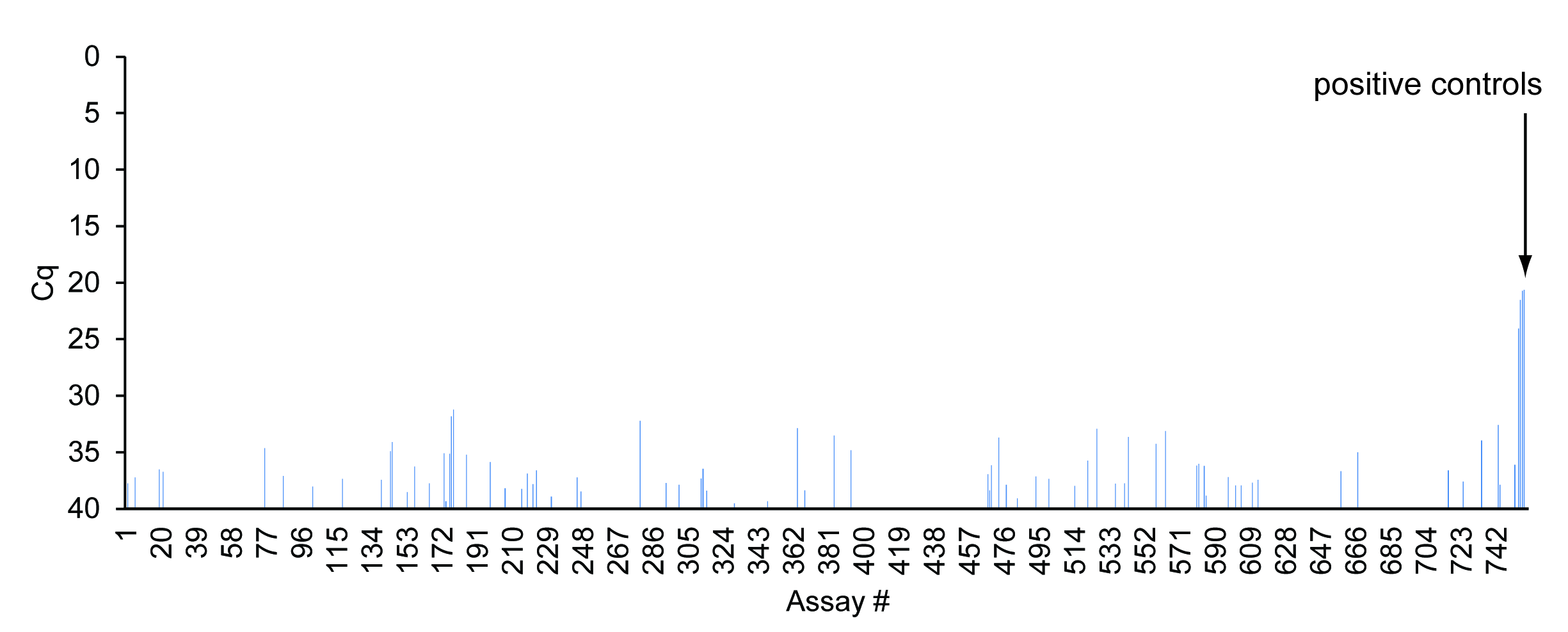

Supplement: Figure S2 — No template control signals for miRCURY array assays. Undetected samples are given the Cq value 40. Positive spike-in controls were included in the water bank sample and are shown on the right of the figure. (TIF) [file pone.0089237.s002.tif]

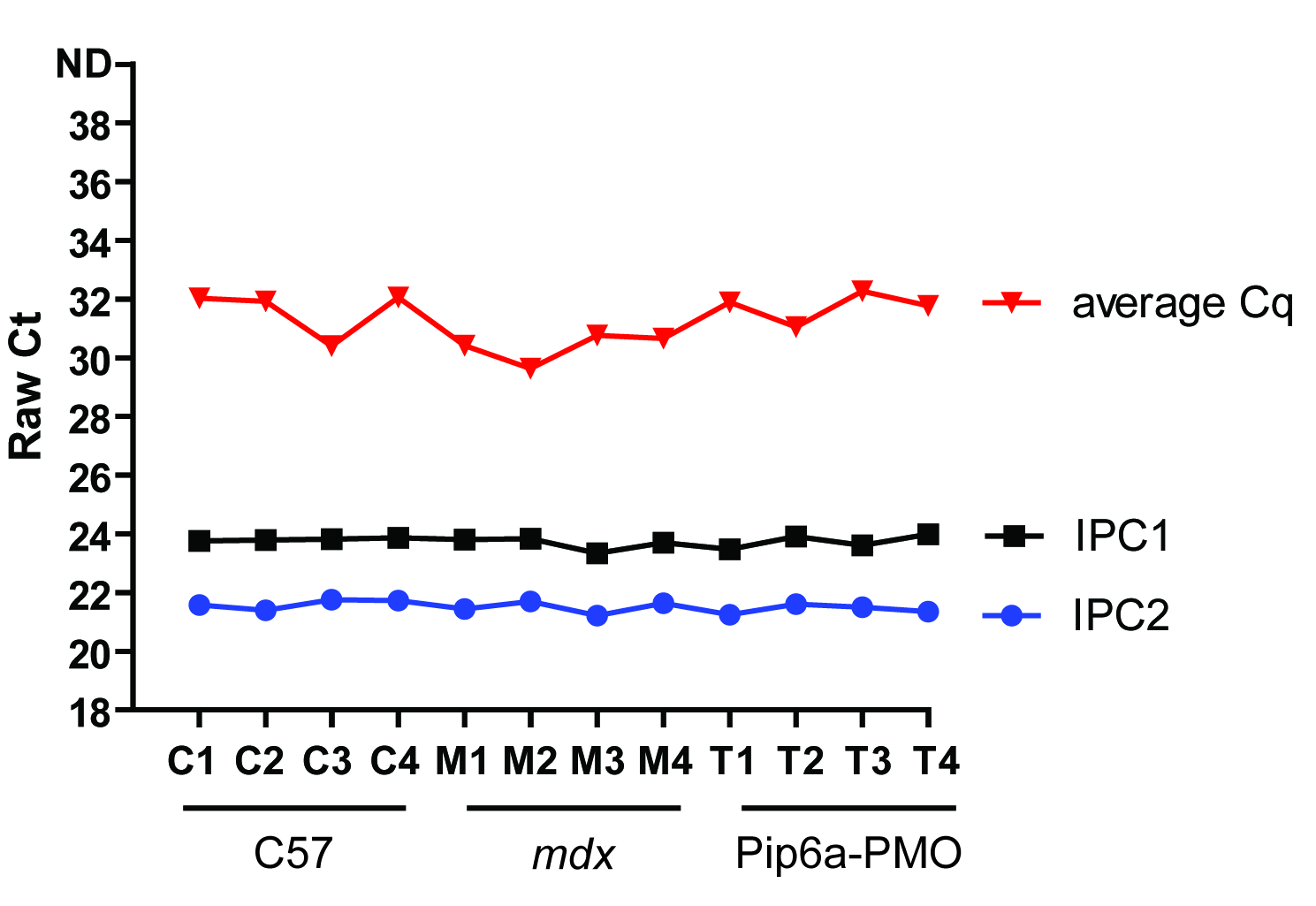

Supplement: Figure S3 — Intra-Plate Controls. Raw Cq values for two intra-plate controls (IPC1 and IPC2) over all samples. The average of all Cqs is shown for comparison. (TIF) [file pone.0089237.s003.tif]
