# Supplementary material for: Assessment of RT-qPCR Normalization Strategies for Accurate Quantification of Extracellular microRNAs in Murine Serum
Source: PLoS One. 2014 Feb 19;9(2):e89237. doi: 10.1371/journal.pone.0089237 (PMC3929707; doi:10.1371/journal.pone.0089237)
Supplement: Table S1 — Summary of datasets used in this study. *single 12.5 mg/kg intravenous dose of Pip6a-PMO which induces efficient dystrophin restoration. **single 12.5 mg/kg intravenous dose of Pip6e-PMO which induces efficient dystrophin restoration. (DOCX) [file pone.0089237.s004.docx]

| **Experiment** | **Platform** | **Experimental Design** |
| --- | --- | --- |
| **Serum miRNA Profiling** | **miRCURY LNA SYBR green RT-qPCR array** (Exiqon)  741 miRNA assays | C57Bl/10 (n=4)  *mdx* (n=4)  Treated *mdx** (n=4)  Harvested age 14 weeks, treated animals injected at 12 weeks |
| **Time Course Study** | **Individual Small RNA TaqMan assays**  (Applied Biosystems)  miR-1, miR-133a, miR-206, miR-22, miR-30a, miR-193b, miR-378, miR-16, miR-223, miR-31, cel-miR-39 | C57Bl/10 (n=44)  *mdx* (n=43)  Treated *mdx*** (n=36)  Harvested at various time points (2 weeks to 48 weeks)  Typically n=4 for each time point |
