# Supplementary material for: Assessment of RT-qPCR Normalization Strategies for Accurate Quantification of Extracellular microRNAs in Murine Serum
Source: PLoS One. 2014 Feb 19;9(2):e89237. doi: 10.1371/journal.pone.0089237 (PMC3929707; doi:10.1371/journal.pone.0089237)
Supplement: Table S2 — List of RT-qPCR Assays used in this study. Assay ID, PCR efficiencies determined using LinRegPCR and R2 values from standard curves are shown. (DOCX) [file pone.0089237.s005.docx]

| **miRNA** | **Assay ID** | **Efficiency** | **R^2^** |
| --- | --- | --- | --- |
| miR-1 | 002222 | 1.868 | 0.9897 |
| miR-16 | 000391 | 1.819 | 0.9983 |
| miR-31 | 000185 | 1.956 | 0.9976 |
| miR-133a | 002246 | 1.853 | 0.9915 |
| miR-206 | 000510 | 1.890 | 0.984 |
| miR-223 | 002295 | 1.724 | 0.9902 |
| cel-miR-39 | 000200 | 1.811 | 0.9896 |
