# Supplementary material for: Assessment of RT-qPCR Normalization Strategies for Accurate Quantification of Extracellular microRNAs in Murine Serum
Source: PLoS One. 2014 Feb 19;9(2):e89237. doi: 10.1371/journal.pone.0089237 (PMC3929707; doi:10.1371/journal.pone.0089237)
Supplement: File S1 — MIQE Compliance Information. (DOCX) [file pone.0089237.s006.docx]

**File S1**

**MIQE Compliance Information**

**Samples**

Serum was harvested from whole blood collected immediately post-mortem as described in Materials and Methods. Samples were separated by centrifugation at 9,000g for 5 minutes at room temperature. Serum samples were flash frozen and stored at -80°C. Samples were processed within 1 year of harvesting.

**Extraction**

For miRCURY array analysis, total RNA was extracted from serum using the miRNeasy® Mini Kit (Qiagen, Crawley, UK).

For Small RNA TaqMan assays extraction was preformed manually using TRIzol LS as described in Materials and Methods. All samples were resuspended in 30 µl of nuclease-free water. No amplification was observed in any RT- or no template controls. Nucleic acid quantification was performed by RiboGreen assay, although this was not used as means for standardizing reactions (see manuscript).

**Reverse Transcription**

For Small RNA TaqMan Assays reverse transcription was performed using the TaqMan MicroRNA Reverse Transcription Kit (Applied Biosystems) using miRNA-specific stem-loop RT primers as according to manufacturer’s instructions with minor modification. Reverse transcription reactions were performed in 20 μl reactions containing 2 µl 10X miRNA RT buffer, 1 µl MultiScribe reverse transcriptase (50 U/µl), 0.15 µl 100 mM dNTP mix, 0.19 µl RNase Inhibitor (20 U/µl) and 1 μl of each RT primer (20X). The remaining volume was made up using nuclease-free water. 5 μl serum RNA was added to each RT reaction (NOTE: RNA input was not standardized between reactions – see manuscript). RT reaction cocktails were incubated at 4°C for 5 minutes, 16°C for 30 minutes, 42°C for 30 minutes and then 85°C for 5 minutes.

**qPCR Target**

All targets are referred to by standard microRNA nomenclature (e.g. mmu-miR-1 or miR-1). Assay IDs listed in **Table S2**.

**qPCR Protocol**

For miRCURY array analysis, 15 µl of RNA was reverse transcribed in 75 μl reactions using the miRCURY LNA™ Universal RT microRNA PCR, Polyadenylation and cDNA synthesis kit (Exiqon). cDNA was diluted 1 in 50 and assayed in 10 µl PCR reactions according to the protocol for miRCURY LNA™ Universal RT microRNA PCR; each microRNA was assayed once by qPCR on the microRNA Ready-to-Use PCR, Rodent panel I and panel II. Additionally, negative controls (no template) were analyzed in parallel. RT-qPCR was performed in a LightCycler® 480 Real-Time PCR System (Roche) in 384 well format. The amplification curves were analyzed using the Roche LC software, both for determination of Cq (by the 2nd derivative method) and for melting curve analysis. PCR efficiencies were determined by LinRegPCR and only assays with efficiencies ranging for 80-110% were included in the analysis. Assays with signal detected on the no template control plate within 5 Cq values of the values detected on the sample plates were also excluded.

For Small RNA TaqMan analysis, qPCR was performed in 20 µl total reaction volume containing 10 µl of 2X TaqMan Gene Expression Mastermix, 1 µl of TaqMan assay (both Life Technologies), 1.33 µl of cDNA and 7.67 µl nuclease-free water per reaction. Universal cycling conditions were used (95°C for 10 minutes (hotstart) and then 40 cycles of 95°C for 15 seconds, 60°C for 1 minute). Real-time amplification was performed on a StepOne Plus thermal cycler (Life Technologies). PCR efficiencies were determined by LinRegPCR and are listed in **Table S2**.

**qPCR Validation**

For miRCURY array analysis, reactions with multiple melt peaks or atypical melting temperatures were excluded from the analysis. For Small RNA TaqMan assays, serial dilutions of cDNA (typically 5 fold) were analyzed. Curves are shown in **Fig. S1** and demonstrate the linearity of the assays over the range of target concentrations measured. R^2^ values are listed in **Table S1**.

**Data Analysis**

Quantification threshold for each assay was determined using the StepOne Software v2.1 (Applied Biosystems) and standardized between plates. Data were analyzed using the Pfaffl method. Specifically, gene expression = E_GOI_^–Cq GOI^/E_REF_^-Cq REF^ where ‘E’ is the pre-determined PCR efficiency, ‘Cq’ is the PCR cycle at which the amplification curve crosses the threshold, ‘GOI’ refers to the gene-of-interest assay and ‘REF’ refers to the reference gene. For technical replicates all qPCR reactions were run in duplicate. Statistical analyses are described in Materials and Methods. For Small RNA TaqMan assays no amplification was observed in any no template controls. For miRCURY arrays, Cqs for no template controls are shown in **Supplementary Figure 2**. Negative amplification was assigned a Cq value of 40. Samples with amplification within 5 cycles of no template control amplification were removed from the analysis. Intra-plate control assay Cq values are shown in **Supplementary Figure 3**.
